# An exploratory study on the perception of the over-the-top (OTT) industry among film and media production major university students: Utilizing the Q methodology

**DOI:** 10.1371/journal.pone.0314035

**Published:** 2025-01-09

**Authors:** Kang Suk Lee, Song Yi Lee

**Affiliations:** 1 Research Institute for Image and Cultural Contents, Dongguk University-Seoul, Seoul, Republic of Korea; 2 Counseling and Coaching, Graduate School, Dongguk University-Seoul, Seoul, Republic of Korea; University of Madeira / NOVA Lincs, PORTUGAL

## Abstract

This study investigates the perceptions of university students majoring in film and media production (FMP) regarding the over-the-top (OTT) industry. We used the Q methodology to achieve this study’s purpose, with 33 Q sets and 22 university students majoring in FMP. The study revealed three perception structures of FMP major university students regarding the OTT industry. Type 1 considered the diversity and accessibility of OTT to be important from the consumer’s perspective, and Type 2 emphasized the importance of improving the quality of content from the producer’s perspective. Even though Type 3 considered the OTT industry an excellent platform, they tended to think of movies in theaters as the final destination. Based on the study’s results, all types thought that the growth and development of the OTT industry would continue. However, the study demonstrated that they need perceptions from a macroscopic viewpoint in areas related to OTT industry technology, education, and systems.

## Introduction

Today’s society has entered an advanced, intelligent age thanks to information and communication technology (ICT). This shift naturally changed the media ecosystem into one centered around platforms [1]. Among platform types, the over-the-top (OTT) streaming platform, which provides broadcast video content in online and offline environments, is in the limelight as a major business item in the relevant industry [2]. Unlike in the past, we can view today’s video content without satellite receivers, antennas, or traditional television (TV) sets—this type of viewing is via OTT services [3]. OTT media companies such as Netflix, Amazon Prime, and Hulu are changing the cooperative relationships between media companies and the structure of the broadcasting industry [4].

In the early days, mainly information technology-related fields used OTT, but its prevalence expanded significantly after the emergence of Netflix [5]. OTT services provide consumer advantages not available before, such as a wider selection of content, easy access, and the flexibility to choose between devices like mobile phones, laptops, tablets, or TV screens [6]. For example, the Squid Game, released by South Korean director Hwang Dong-hyuk in 2021 on Netflix, an international OTT streaming platform, goes beyond the popularity of the video. It has become a prime example of a new video content culture, generating diverse memes and producing and distributing one-source-multi-use (OSMU) products in the online space [7]. The emerging platform culture from the OTT industry offers positive aspects by exposing people to new cultures and values. However, from an industry standpoint, it presents a problem with its monopolistic market structure, hindering balanced development by preventing the entry of more diverse business entities [8]. Specifically, the imbalance problem in South Korea is more pronounced as OTT platforms widely distribute many box office hits, incorporating elements of the country’s culture and garnering international acclaim.

The size of the OTT market is growing, and the quality of content is steadily improving. However, researchers continue to highlight the problem of the market’s monopoly [1]. Regardless, the OTT industry creates economic effects and produces numerous internationally acclaimed works. While trends in global research on the OTT industry raise concerns that the emergence of global OTT platforms could shrink broadcasting sectors and allow large global companies to dominate, there is also a positive view that the growth of the OTT industry can catalyze innovation in the broadcasting and communications industry [9].

Researchers in South Korea are studying the perceptions of university students majoring in fields related to the OTT industry (i.e., the primary consumer base) regarding sustainable growth measures for OTT industry development [10, 11]. When discussing the future development potential of a certain industry, collecting the opinions of university students majoring in areas related to the industry is an activity with an important meaning [12]. Scholars use this approach because this group serves as the foundation for connecting industry and academia—university students can address industry challenges creatively since they receive specialized training in related industries [13].

However, in exploring problems related to the OTT industry and studying areas for improvement in related industries, studies conducted with university students majoring in film and media production (FMP) closely related to OTT are still insufficient. Therefore, understanding the perceptions of prospective human resources, specifically university students majoring in relevant fields can significantly inform the industry’s future services. Their views on the industry’s developmental direction are fundamental and essential.

## Literature review

The rise of the Internet and smart devices has enabled the public to access video content anytime, anywhere [14]. OTT services, which generate traffic over wired and wireless networks, offer various services such as communication, real-time entertainment, file-sharing, and web browsing. The nature of this traffic varies based on its purpose and type [3]. Therefore, transmitting information through the Internet is sometimes called an OTT service. For example, Busson et al. [15] broadly defined OTT service as providing videos or audio over the Internet. Kraemer and Wohlfarth [16] also defined OTT service as providing video content to various terminals via the Internet. Therefore, an OTT service is a platform that provides a variety of video-based content in situations where equipment or devices that rely on traditional set-top boxes are not necessary.

We can divide OTT services depending on their revenue models: (1) subscription-type revenue models such as Netflix, Disney, Wave, etc., (2) advertising revenue models in which the users obtain the right to view content when they watch certain advertisements, such as YouTube, and (3) a purchase-based (transaction) revenue model in which users pay for the content they want to watch through Internet protocol television (IPTV), etc. The subscription-type models account for the highest proportion [8, 17], with the largest operator, Netflix, which has over 280 million members worldwide. According to a 2022 survey, Netflix recorded a 45.2% share of the OTT market in the United States [18]. In addition, the size of the OTT market, led by international OTT service platforms such as Amazon Prime Video, Disney Plus, and Apple TV Plus, is hitting an average annual growth rate of 28.7% as of 2023 [19]. The OTT market has become substantial, with a global market size worth about $87.99 billion in 2022 [20]. Therefore, comprehending consumer usage patterns and perceptions of OTT services is crucial for the rapid development and growth of the industry.

Among studies conducted from a consumer perspective, Menon [21] investigated the relationships between diverse uses and gratifications (U&G), subscriptions, and intentions to continue with various OTT streaming platforms in India. They identified eight major factors in OTT use: convenient navigability, binge-watching, entertainment, relaxation, social interaction, companionship, voyeurism, and information seeking. In addition, Sujith and Sumathy [22] looked at satisfaction with OTT and found that perceived usefulness, perceived ease of use, and overall perception largely affect satisfaction with OTT. According to Nijhawan and Dahiya [6], the flexibility to access a variety of content through personal devices such as smartphones and tablets allows one to watch what one wants freely. Also, a study in India found that the preceding flexibility contributed to popularization in cities with high purchasing power. Previous studies also indicate that OTT operators’ common strategies include localization, partnership, content differentiation, profit enhancement, and service optimization [4]. In addition, study findings indicate that people are enthusiastic about movies released on OTT platforms at certain hours. However, when watching movies, they prefer to watch them in theaters rather than on OTT platforms [23].

Among studies related to the development of the OTT industry, Karad et al. [24] revealed that various factors such as geographical locations, movie genres, the levels of recognition of actors and directors, and the history of the director’s past works are important in the film production process. According to Shim and colleagues [25], to develop the OTT industry, OTT service providers can expect to increase the conversion rate from free users to paid users and increase sales by effectively targeting free users in a fiercely competitive environment. Therefore, it is meaningful to focus on understanding consumers’ OTT use patterns and demographic variables to find profitable niche markets. Additionally, exploring content strategies entails considering which services to design based on content implementation. Thus, studies on the OTT industry mainly focus on the relationship between variables such as consumers’ cognitive structures and intentions to view OTT services [26, 27].

Despite existing studies examining the development of the OTT industry and exploring factors related to its growth and user satisfaction, there is a notable lack of research delving into the perspectives of university students majoring in relevant fields who have the potential to become consumers and producers in this field. According to Zhang and Luo [28], consumers should use media marketing to extend the life cycle of intellectual property and promote sustainable brand development. Therefore, this exploratory study identifies the perception structure of university students majoring in FMP regarding the OTT industry and comprehensively discusses the problems and future direction of South Korea’s OTT industry. Thus, this study’s research questions are:

RQ1: What are the OTT industry types perceived by film and media production major university students?
RQ2: What are the characteristics of the OTT industry types perceived by film and media production major university students?

## Study method

**Fig 1:**
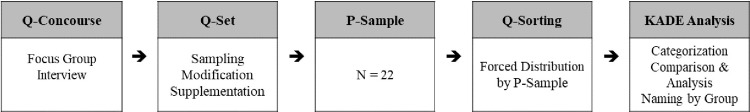
Q methodology process.

William Stevenson developed the Q methodology in the 1930s to provide a scientific framework for examining subjectivity [29]. The Q methodology enables investigating how people in a certain culture perceive a certain topic. Through the exploration process, researchers divide perceptions of certain topics into different groups and types to examine the characteristics of those types. The Q methodology is not a study method for statistical generalization with a large number of participants; it is a way to uncover the cognitive structure of subjects related to a certain topic. In other words, Q methodology is the exploration of practical reasoning by linking the generalization of phenomena to a specific group rather than making statistical inferences about the entire population [30]. The core of Q methodology is the perceptions of study participants. In the study procedure, the researcher first selects a set of representative perceptions from the Q concourse, which is the totality of perceptions of the topic. Next, the researcher selects participants to perform Q sorting, where they classify their perceptions. Finally, the researcher analyzes and interprets the collected data. Fig 1 illustrates this process.

In addition, we obtained approval from the Dongguk University Institutional Review Board for Bioethics (approval number: DUIRB-202310-17) on October 18, 2023. Following IRB regulations, we thoroughly explained the study’s purpose and procedures before engaging the participants in the study. We also received signed written consent forms from the students.

### Q concourse

A Q concourse represents the collective thoughts of people within a certain culture on a topic of interest. In this study, since we’re examining perceptions of the OTT industry by university students majoring in film and media production, we transform their thoughts into statements, forming the Q concourse. This study created a Q concourse through focus group interviews. We used purposive sampling to select eight university students majoring in FMP (females, n = 5; males, n = 3) to participate in the focus group interviews on November 14, 2023. The sample included two students from each of the four years, majoring in film and media production. Before the interview, we comprehensively explained the purpose and procedures of the study.

After agreeing to participate, the students signed written consent forms with the researchers present. The researchers collected the signed written consent forms for retention purposes. The interviews took about an hour, and we gave 20,000 won to each student as a token of gratitude. The semi-structured questions used in the interviews to construct the Q concourse consisted of queries such as “What do you think about the OTT industry,” “What are the positive aspects of the OTT industry,” and “What are the negative aspects of the OTT industry?” After recording and transcribing the interview contents, we created 104 statements.

### Q set

A Q set is a representative list of statements from the Q concourse. From an original set of 104 statements, researchers selected 50 they agreed to be representative and comprehensive. Following this, the researchers deliberated on choosing content closely aligned with the study’s topic while maximizing diversity. Subsequently, they selected 33 statements, shown in Table 1.

**Table 1:** Statements.

No.	Statements
1	Being able to rewatch the content easily is a huge strength of OTT.
2	With the diversity of OTT videos, I think a lot of work is available for people in related majors.
3	I think the prospects for the OTT industry are bright.
4	The rise of OTT has made getting the resources I need to study in school easier.
5	I think OTT has helped to reduce the use of illegal content.
6	I think that OTT has advantages in terms of accessibility.
7	I’m impressed that the diversity of OTT content allows it to cater to a wide range of people.
8	OTT allows producers to plan content without being limited due to demand.
9	I think the movie theater industry has the potential to be negatively affected because of the OTT industry.
10	I think one of the positive aspects of OTT is that it’s subdivided by taste rather than for the general public.
11	I wish there were a curriculum that analyzes OTT content and platforms.
12	As OTT made videos more accessible, people who would never have watched videos have now become viewers.
13	I think it’s more important to be able to do that because of the importance of artistic quality.
14	With easy access to international media content through OTT, the sense of distance between countries disappears.
15	I think that it is an advantage when it comes to choosing a career in OTT because I have a lot of options.
16	I find it interesting that OTT can create various content compared to movies.
17	I think it is great that producers and actors have a wider range of choices in terms of opportunities.
18	I aim to create content that will ultimately be shown on the big screen in cinemas, leveraging OTT as a stepping stone.
19	I feel that OTT tends to lean towards commerciality.
20	I hope that the film industry can develop independently of OTT platforms.
21	OTT platforms tend to provide more violent and provocative content.
22	I believe there should be increased regulation concerning age restriction.
23	There needs to be a consensus on aligning regulations with foreign standards.
24	Due to the high growth potential of domestic OTT businesses, I believe it could present many opportunities.
25	The policies regarding OTT are vague, making it difficult to make a clear career plan.
26	I’m not particularly interested in OTT, as I believe it might soon die out with the emergence of other media platforms.
27	I aspire to venture into the international OTT industry, which allows for various opportunities.
28	I see OTT as a necessary step to enter the film industry.
29	The curriculum should include a course on the introduction to OTT for relevant majors.
30	I believe that maintaining the artistic quality of content on OTT platforms is crucial for the sustainability of the industry.
31	Given that OTT requires an understanding of new technologies, preparation in this regard is necessary.
32	Viewers who prioritize artistic quality might have less interest in OTT.
33	Since diverse age groups can watch movies or dramas through OTT, it allows for creating common interests across generations.

### P sample

P samples are the persons related to the topic; in this study, P samples are the university students majoring in film and media production. Since the purpose of the Q methodology is not to generalize but to examine the diversity of opinions, researchers do not select P samples randomly. Instead, they intentionally select subjects related to the study [31, 32], ranging from 10 to 100 participants [33]. The Q methodology can produce powerful results even with few samples [32]. Table 2 shows 22 university students majoring in film and media production who participated in this study. The Q sorting was done from November 30 to December 11, 2023, which took about an hour for each participant. We gave 10,000 won to each student as a token of gratitude.

**Table 2:** Factor matrix with defining sorts flagged.

P sample	Gender	Year	Level	Factor 1		Factor 2		Factor 3	
1	Female	4	High	0.7374	Flagged	0.4352		0.0698	
2	Male	1	High	0.2538		0.6878	Flagged	−0.0329	
3	Male	2	High	0.1519		0.1231		0.338	Flagged
4	Female	3	High	0.7155	Flagged	0.2272		0.0187	
5	Female	3	High	0.4048		0.4116	Flagged	0.3929	
6	Female	2	Medium	0.5139	Flagged	0.3662		−0.0189	
7	Female	2	High	0.646	Flagged	0.2477		0.1096	
8	Female	2	Medium	0.4523		−0.0179		0.6707	Flagged
9	Female	3	High	0.6697	Flagged	0.2737		0.2478	
10	Female	3	Medium	0.6058	Flagged	0.3045		0.3883	
11	Female	2	Medium	0		0.5471	Flagged	0.2364	
12	Female	3	High	0.7785	Flagged	0.1338		0.2106	
13	Female	4	High	0.2191		0.6187	Flagged	0.4329	
14	Male	3	Medium	0.7689	Flagged	−0.0644		0.0219	
15	Female	2	Medium	0.3765	Flagged	−0.2789		0.1763	
16	Female	4	Medium	0.2418		0.6927	Flagged	−0.1771	
17	Female	4	Low	0.0786		0.7061	Flagged	0.1918	
18	Female	4	Low	−0.0652		0.0596		0.6312	Flagged
19	Female	2	Medium	0.6453	Flagged	0.3714		0.4654	
20	Female	4	Medium	0.1907		0.1322		0.7851	Flagged
21	Male	3	Medium	0.0005		0.5635	Flagged	0.2853	
22	Female	2	High	0.6539	Flagged	−0.1586		0.1917	
% Explained Variance				24		16		12	

### Q sorting and data analysis

**Fig 2:**
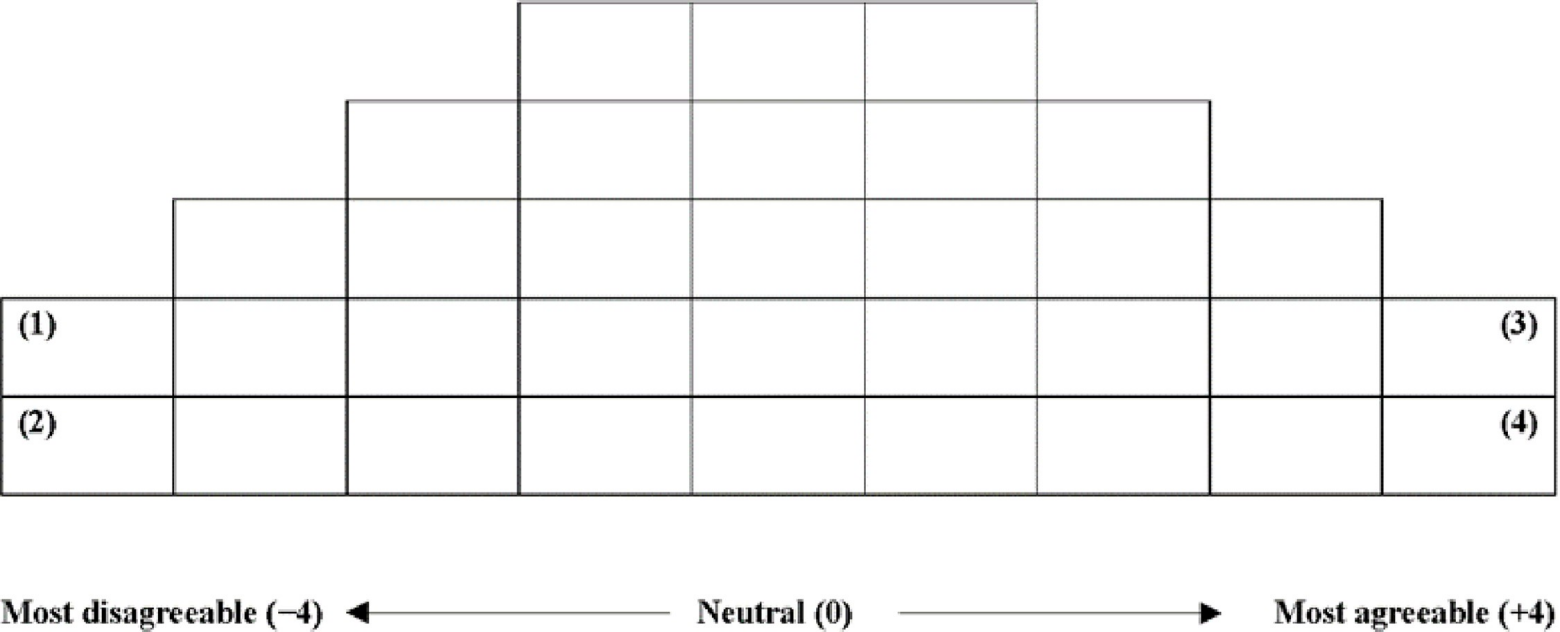
Q sorting grid.

Q sorting involves assessing the extent to which P samples (study participants) agree or disagree with specific statements, allowing researchers to categorize their perspectives on media-portrayed violence for comparative analysis. Additionally, when the participants underwent Q sorting, we asked them to indicate their intention to enter the OTT industry by responding with high, medium, or low. Q sorting involved arranging statements within a Q-grid based on varying degrees, as illustrated in Fig 2. We used the KADE program to analyze the data derived from the Q sorting procedure.

Upon obtaining Z-scores for each factor, we focused on statements with Z-scores reaching an absolute value of 1.000 or higher, indicating their significance for further analysis. Additionally, we examined detailed explanations provided by P samples regarding their rationale behind selecting two strongly agreeable and two strongly disagreeable statements, aiding in additional interpretation.

## Results

### Analysis of results

We derived three factors. The eigenvalues for the individual types were 24 for Type 1, 16 for Type 2, and 12 for Type 3, and the cumulative variance was 52.

### The characteristics of the factors

#### Type 1: A type that recognizes the strengths of accessibility and diversity

Eleven students belong to Type 1: seven university students responded ‘high’ and four responded ‘medium’ regarding their intention to enter the OTT industry.

As per Table 3, Type 1 showed strong agreement with S6: “I think that OTT has advantages in terms of accessibility,” S7: “I’m impressed that the diversity of OTT content allows it to cater to a wide range of people,” and S4: “The rise of OTT has made getting the resources I need to study in school easier.” They strongly disagreed with S28: “I see OTT as a necessary step to enter the film industry” and S9: “I think the movie theater industry has the potential to be negatively affected because of the OTT industry.”

**Table 3:** Factor scores for Factor 1.

No.	Statement	Z−score
6	I think that OTT has advantages in terms of accessibility.	1.727
1	Being able to rewatch the content easily is a huge strength of OTT.	1.667
21	OTT platforms tend to provide more violent and provocative content.	1.372
7	I’m impressed that the diversity of OTT content allows it to cater to a wide range of people.	1.342
4	The rise of OTT has made getting the resources I need to study in school easier.	1.328
22	I believe there should be increased regulation concerning age restriction.	1.163
32	Viewers who prioritize artistic quality might have less interest in OTT.	−1.020
9	I think the movie theater industry has the potential to be negatively affected because of the OTT industry.	−1.056
18	I aim to create content that will ultimately be shown on the big screen in cinemas, leveraging OTT as a stepping stone.	−1.117
25	The policies regarding OTT are vague, making it difficult to make a clear career plan.	−1.395
26	I’m not particularly interested in OTT, as I believe it might soon die out with the emergence of other media platforms.	−1.691
28	I see OTT as a necessary step to enter the film industry.	−1.842

P1, who belongs to Type 1, said, I like that OTT allows me to watch dramas I used to enjoy or movies I missed in theaters. It is easily accessible, and I can watch it whenever it’s convenient. Its strength lies in the flexibility it offers consumers to watch according to their circumstances.

Also, P12 said, “I think that OTT’s effect on the overall film industry will gradually grow. OTT’s strength is that everyone can watch classic movies anytime, anywhere. Thanks to various OTT platforms, classic movies are relatively more easily found.” In addition, P7 said, “Because there is less risk of investment in OTT works compared to works shown in theaters, people are creating many minor and diverse works, satisfying the needs of various people through the preceding.”

Type 1 thinks that the outlook for the OTT industry is bright, focusing on content that can satisfy the needs of a variety of people and the ease of access to such content. Type 1 also believes that the film industry, screened in theaters, will not retreat due to OTT.

#### Type 2: A type aroused about high-quality content

Seven university students belong to Type 2. Three responded ‘high,’ three ‘medium,’ and one ‘low’ regarding their intention to enter the OTT industry.

As shown in Table 4, Type 2 strongly agreed with S19: “I feel that OTT tends to lean toward commerciality,” S30: “I believe that maintaining the artistic quality of content on OTT platforms is crucial for the sustainability of the industry,” and S20: “I hope that the film industry can develop independently of OTT platforms.” They strongly disagreed with S32: “Viewers who prioritize artistic quality might have less interest in OTT” and S8: “OTT allows producers to plan content without being limited due to demand.”

**Table 4:** Factor scores for Factor 2.

No.	Statement	Z−score
1	Being able to rewatch the content easily is a huge strength of OTT.	1.943
19	I feel that OTT tends to lean toward commerciality.	1.512
6	I think that OTT has advantages in terms of accessibility.	1.42
30	I believe that maintaining the artistic quality of content on OTT platforms is crucial for the sustainability of the industry.	1.294
20	I hope that the film industry can develop independently of OTT platforms.	1.118
33	Since diverse age groups can watch movies or dramas through OTT, it allows for creating common interests across generations.	−1.097
18	I aim to create content that will ultimately be shown on the big screen in cinemas, leveraging OTT as a stepping stone.	−1.264
8	OTT allows producers to plan content without being limited due to demand.	−1.498
26	I’m not particularly interested in OTT, as I believe it might soon die out with the emergence of other media platforms.	−1.628
32	Viewers who prioritize artistic quality might have less interest in OTT.	−1.633
28	I see OTT as a necessary step to enter the film industry.	−1.775

P16, who belongs to Type 2, said, “I think OTT has nothing to do with artistic quality and is more focused on pursuing commercial interests.” Also, P17 said, “I think the popularity of OTT original content is decreasing, and that the reason is the low quality of works and poor stories. I also think that dealing with provocative and violent topics biases toward commercialism.” P11 said, “The development of OTT is infringing on the development of movies.”

Type 2 expressed concerns about the OTT industry’s bias toward commercialism and recognized the need to improve the quality of content for development. They think the quality of work is important, but consumers do not seriously consider it.

#### Type 3: A type that recognizes incomparable quality of work and content excellence

Four students belong to Type 3. Among them, one responded ‘high,’ two responded ‘medium,’ and one ‘low’ regarding their thoughts on entering the OTT industry.

As outlined in Table 5, Type 3 strongly agreed with S7: “I’m impressed that the diversity of OTT content allows it to cater to a wide range of people” and S12: “I think as OTT made videos more accessible, people who would never have watched videos have now become viewers,” S16: “I find it interesting that we can create various content compared to movies,”and S18: “I aim to create content that will ultimately be shown on the big screen in cinemas, leveraging OTT as a stepping stone.” They strongly disagreed with S31: “Given that OTT requires an understanding of new technologies, preparation in this regard is necessary” and S11: “I wish there were a curriculum that analyzes OTT content and platforms.”

**Table 5:** Factor scores for Factor 3.

No.	Statement	Z−score
1	Being able to rewatch the content easily is a huge strength of OTT.	1.573
30	I believe that maintaining the artistic quality of content on OTT platforms is crucial for the sustainability of the industry.	1.566
7	I’m impressed that the diversity of OTT content allows it to cater to a wide range of people.	1.455
18	I aim to create content that will ultimately be shown on the big screen in cinemas, leveraging OTT as a stepping stone.	1.233
12	As OTT made videos more accessible, people who would never have watched videos have now become viewers.	1.079
16	I find it interesting that one can create a variety of content compared to movies.	1.026
3	I think the prospects for the OTT industry are bright.	1.023
24	Due to the high growth potential of domestic OTT businesses, I believe it could present a lot of opportunities.	−1.011
25	The policies regarding OTT are vague, making it difficult to make a clear career plan.	−1.182
23	There needs to be a consensus on aligning regulations with foreign standards.	−1.196
11	I wish there were a curriculum that analyzes OTT content and platforms.	−1.358
31	Given that OTT requires an understanding of new technologies, preparation in this regard is necessary.	−1.542
32	Viewers who prioritize artistic quality might have less interest in OTT.	−1.805
26	I’m not particularly interested in OTT, as I believe it might soon die out with the emergence of other media platforms.	−2.089

P20, who belongs to Type 3, said, I think that the advantage of OTT is that there are many works with excellent quality of work, although they are OTT content, and viewers can choose and view the content that fits their taste among various content. Since the probability for viewers to choose the content increases when the quality of the work is excellent, the quality of the work is the most important factor we should not lose.”

In addition, P8 said, “Because OTT covers all movies, dramas, and entertainment, I don’t think media other than OTT will be popular.” Along with this, P3 said, “OTT platforms have helped reduce the widespread use of illegal content in the country. Due to this, word-of-mouth has become more important, and there is now a greater emphasis on artistic quality over the sheer number of works.”

Type 3 believes that OTT has diverse content, and the content has quality work. They recognize that these strengths are superior to any other media platforms. However, although they are aware of the excellence of the OTT industry, they thought they would rather work for movies that are shown in theaters rather than continue working in this field.

Table 6 depicts the consensus statements resulting from this study. The study’s participants agreed with S1: “‘Easy to watch again is a big strength of OTT” and disagreed with S26:”I’m not particularly interested in OTT, as I believe it might soon die out with the emergence of other media platforms.”

**Table 6:** Consensus statements.

No.	Statement	Factor 1 Z-score	Factor 2 Z-score	Factor 3 Z-score
1	Being able to rewatch the content easily is a huge strength of OTT.	1.667	1.943	1.573
5	I think OTT has helped to reduce the use of illegal content.	0.430	−0.040	−0.150
13	I think it’s more important to be able to do that because of the importance of artistic quality.	−0.776	−0.871	−0.342
15	I think that it is an advantage when it comes to choosing a career in OTT because I have a lot of options.	0.310	−0.160	−0.410
24	Due to the high growth potential of domestic OTT businesses, I believe it could present many opportunities.	−0.360	−0.756	−1.01
26	I’m not particularly interested in OTT, as I believe it might soon die out with the emergence of other media platforms.	−1.691	−1.628	−2.089

## Discussion

This study explored the over-the-top (OTT) industry perceptions of university students majoring in film and media production. Based on the study’s results, we found three types. Type 1 recognizes the strengths of accessibility and diversity; Type 2 is excited about the high-quality content, and Type 3 acknowledges the incomparable quality of work and content excellence.

Type 1 noted accessibility and diversity, which are the strengths of the OTT industry. This observation is similar to Pekpazar et al.’s [17] study to develop OTT. It indicates that accessibility and customization are crucial in usability evaluation, as it impacts the readability of texts and subtitles on OTT platforms. In addition, Type 1 shows characteristics similar to a study by Nijhawan et al. [6], finding that flexibility promotes the spread of OTT. They found that OTT satisfied the needs of consumers and perceived that OTT is in an environment where content fits various audiences, and access to the content is easy. Many Type 1 students show the characteristic of wanting to enter the OTT industry and consider the development of the OTT industry from the position of consumers. They also think of the possibility of development thanks to consumers’ advantages.

Priya et al. [34] revealed that OTT platforms influence consumer loyalty and usage intentions. This finding aligns with Type 1’s view that the accessibility of OTT platforms is important for their growth. In this context, it is crucial to incorporate content that promotes an understanding of OTT platforms in the film and media studies curriculum. As OTT platforms have established themselves as the main channels for consuming diverse media content such as films, dramas, and documentaries, students need to understand the intrinsic characteristics of OTT and how it enables the production, distribution, and consumption of media.

The curriculum should include a fundamental understanding of OTT platforms’ operational mechanisms and business models, allowing students to learn how platforms provide content and generate revenue, and the differences between subscription-based and ad-based models. By exploring the impact of technical elements, such as content recommendation algorithms, on consumer experience and loyalty, students will gain insights into how platforms offer personalized services to users. Moreover, education on the technological aspects of OTT platforms should be integrative OTT services provide content and operate through technological foundations such as cloud computing, data analytics, artificial intelligence, and big data utilization. Therefore, students must learn and understand these technological elements.

Through instruction on the principles of streaming technology, server infrastructure, and network optimization, students will understand the technologies OTT platforms use to deliver high-quality services to consumers. Additionally, courses that analyze the trends in the global OTT market and the competitive landscape among various platforms are essential. These courses will enable students to understand OTT’s impact beyond being merely a media consumption platform and study the characteristics of content consumed across different cultural contexts. Such education equips students with the essential knowledge and skills for working in future OTT-related roles within the media industry. Thus, a comprehensive education covering various areas, such as business models, technological aspects, and global trends, is essential for a deep understanding of OTT platforms. This education will enable film and media studies students to understand OTT platforms holistically and develop the competencies needed to thrive in the future media industry.

Type 2 students have a moderate degree of thought to enter the OTT industry. They believe that OTT content is overly structured for commercial purposes and feel that people need greater awareness about OTT. Although they agree with the strengths of the OTT industry, they believe that the fundamental high-quality content and the quality of work of the media must be continuously maintained and developed. However, they did not think that viewers who think of the quality of work would be less interested in OTT. They believe that since other factors also affect OTT viewing from the consumer’s perspective, producers should be concerned about the quality of work to develop OTT.

In addition, Type 2 strongly believes content should prioritize quality to engage consumers and boost commercial success. Developing content with consideration for genres, tastes, and quality that aligns with consumer preferences is an important element in the OTT industry. This belief supports the study’s findings that killer content greatly affects users’ choice of OTT services and that services can replace and complement each other to simultaneously satisfy the tastes of various content genres appropriate for the scale and characteristics [35].

In developing the OTT industry, the efforts and roles of producers are crucial factors. Type 2 emphasizes this changing role of producers, suggesting that proper regulation is essential. As OTT platforms become increasingly prominent in the global media landscape, the regulations of each country can significantly influence the direction and success of the industry. In particular, the study by Bilbil [36] underscores the importance of regulating OTT from a multi-dimensional perspective, arguing that authorities should not limit regulations to content or platforms but encompass a broader standpoint.

Advancing the OTT industry requires discussing regulations that address the roles of content creators, distributors, and platform operators rather than focusing solely on content regulation. Bilbil’s [36] study highlights that OTT regulations should go beyond content quality and ethics to include consumer rights protection, data privacy, copyright issues, and fairness in content distribution. Such comprehensive regulation can foster a sustainable environment for the long-term growth of the OTT industry—establishing an appropriate regulatory framework is critical.

Studies on international cases related to OTT regulation demonstrate that countries adopt different approaches depending on their situation. For instance, Isa et al. [37] analyzed OTT regulatory cases from countries like Australia, China, and Turkey, comparing the impact of each country’s regulations on the OTT industry. They found that Australia has adopted regulations focusing on managing content quality and protecting consumers, while China strongly regulates OTT content from national security and ideological control perspectives. On the other hand, Turkey has implemented policies to prevent platform monopolies and protect domestic content. These cases provide valuable insights for discussions on OTT regulation in South Korea.

In South Korea, OTT regulation focuses particularly on protecting children and adolescents, emphasizing the role of parents. According to Isa et al. [37], South Korea highlights the importance of parental guidance and control in regulating OTT content that could negatively impact children, suggesting the need for self-regulation to protect minors. The study shows that collaboration between the government and platforms is necessary to provide parents with tools to manage content and create a safe environment for children. In addition, Kim and Kim [38] criticize the approach of regulating OTT platforms in the same way as traditional media, calling it unrealistic and convenient. Unlike traditional media, OTT operates in real-time in the global market, allowing consumers to watch content conveniently, creating a different ecosystem from traditional media. Therefore, applying existing broadcasting laws or media regulations to OTT may hinder the industry’s development. The study stresses the need for a new regulatory framework tailored to the OTT industry.

These discussions on OTT regulation highlight the importance of understanding the overall situation and designing regulations that promote the positive development of the OTT industry. Regulation plays a key role in helping OTT platforms and content creators operate responsibly, enhancing competitiveness and ensuring fairness in the market. Accordingly, South Korea must also engage in in-depth discussions on domestic and international OTT industry regulations and develop regulatory measures that consider competitiveness in the global market.

Regulators should discuss OTT regulation, including content quality and ethics, and from a broader perspective, encompassing platform data management, privacy protection, copyright issues, and other macro aspects. This approach means that regulation should go beyond content censorship and consider platform operation and technological advancements. Thus, the industry needs continuous attention and research on OTT platform regulation globally and how to proceed with South Korea’s OTT regulations. Therefore, regulators should adopt a more macro and multi-dimensional regulatory approach to foster positive OTT industry development, referencing international cases. Domestic regulations should also gradually improve in this direction, enabling producers to adapt to the changing regulatory environment and contribute to the industry’s growth by delivering better content and services.

Type 3 expressed various opinions on entering the OTT industry but shared a strong perception that it will continue to grow, supported by its exceptional platforms. Nevertheless, they were more interested in producing content for screening at intrinsic movie theaters than in the OTT industry. This perspective indicates that the development of OTT is not an unconditional career path for students majoring in film and media production (FMP). Therefore, they consider the OTT industry an intermediate step for the traditional film industry for theater screening. They believe the theater-centered film industry is important, and in terms of diversity, their thoughts are in the same vein as the study findings of Sun Ah Kim [1]. That study also found a media convergence environment and that producers should recognize the importance of the offline film industry centered on theaters. They found that efforts to protect the offline film industry, including revising the law, are necessary.

In addition, while Type 3 looked at the development of the OTT industry from a very positive perspective, they did not seem to consider the introduction of curriculums into university education or preparation for improving technology’s ability as important. Eun-Hye Lee [39] said that for the development of South Korea’s entertainment industry, a field-oriented curriculum that closely links industry and education and the cultivation of talents equipped with practical capabilities are necessary. Type 3 must recognize the preceding and supplement realistic capabilities for the OTT industry.

According to the study by Varghese and Chinnaiah [40], modern viewers are continuously seeking original content, connected experiences, and interactive entertainment, and they are willing to pay extra for immersive experiences offered in theaters or through OTT platforms. These findings suggest that theaters and OTT platforms are not in a competitive relationship but have the potential to coexist in the long term. Audiences are increasingly desiring the convenience of OTT platforms and the immersive experience of theaters, indicating that these two mediums can play complementary roles. Thus, this research highlights the need for students with perspectives similar to those of Type 3 to engage in deeper reflection. Type 3 students view OTT platforms positively but maintain a strong interest in the theater industry, which may lead to a relative disinterest in OTT-related curricula.

However, for the continued development of the theater industry, it is essential to understand the characteristics of OTT platforms and formulate strategies to complement them. Varghese and Chinnaiah’s [40] findings show that modern audiences do not view OTT platforms and theaters as substitutes but as complementary mediums offering different experiences. There is ample room for these two industries to coexist. While OTT platforms are convenient and accessible anytime and anywhere, theaters still offer the unique, immersive experience of large screens and sound systems. Audiences tend to consume both services in different situations, suggesting that OTT platforms and theaters can thrive by leveraging their strengths to grow together rather than conflict.

If Type 3 students are highly interested in the theater industry but less engaged with OTT-related education, this could pose a challenge. For the theater industry to grow, it is necessary to understand the innovation and growth within the OTT industry and develop complementary strategies. The accessibility of OTT, personalized content recommendations, and access to the global market offer valuable lessons for the theater industry. For example, theaters can implement personalized marketing strategies based on data-driven audience analysis or offer immersive viewing experiences tailored to specific genres or themes. OTT platforms and theaters can form a complementary relationship, meeting the diverse needs of audiences without competing.

While OTT provides audiences with easy access to content anytime, theaters offer differentiated experiences through special events and immersive viewings. This situation implies that the theater industry can attract more audiences by embracing new content consumption trends from OTT platforms and offering differentiated experiences based on these trends. Therefore, understanding the characteristics of the OTT industry through educational approaches is essential for advancing the theater business. Students should gain insights into how OTT and theaters can complement each other, thus acquiring a more comprehensive understanding of the film industry. By learning about streaming technologies, content distribution methods, and consumer behavior analysis from OTT platforms, theaters can develop the knowledge and strategies needed to offer audience-tailored experiences. For example, theaters could hold online premieres alongside offline screenings or plan theater events in collaboration with OTT platforms to maximize synergy between the two mediums. Rather than competing, it is more desirable for the theater and OTT industries to pursue complementary growth. For instance, theaters could re-screen popular content on OTT platforms or expand successful films into versions or spin-offs on OTT platforms. This collaborative development would allow audiences to enjoy both viewing experiences, fostering overall industry growth and creating new business opportunities.

The study by Varghese and Chinnaiah [40] indicates that modern audiences recognize the potential for OTT platforms and theaters to coexist, with both industries capable of developing complementarily. Instead of focusing solely on the theater industry, Type 3 students need to understand the characteristics of the OTT industry and develop strategies to integrate it with the theater industry. Achieving this requires an expanded curriculum that supports the fusion of OTT and theater development, allowing students to play a key role in shaping the film industry’s future.

All three types commonly agreed that the strength of OTT is its ease of rewatching, and regardless of their intentions to enter the OTT industry, they commonly disagreed with the opinion that the popularity of OTT will soon fade due to the emergence of other media. All types recognize the strengths of OTT and show a positive view of its growth potential. Thus, based on this study’s results, we offer the following suggestions.

First, university students majoring in film and media production should be able to look at the OTT industry from a macro perspective. In this study, when selecting statements to examine the students’ perceptions of the OTT industry, although there were educational and institutional statements, no type showed the preceding characteristics. Therefore, these students do not recognize the OTT industry from a more macroscopic perspective. A study [41] with a group of experts to examine the competitiveness factors necessary for domestic OTT platforms to advance overseas found that cooperation with local networks and telecommunications companies is important, along with OTT-related regulatory policies, cultural barriers, and economic levels of the countries they are entering. This finding shows the difference in the areas that university students and experts mainly recognized for developing the OTT industry. The finding also indicates that film and media production major university students must be interested in content and areas in related systems and laws to contribute to the OTT industry’s development.

Second, the FMP majors’ ‘quality of work’ concept requires a clear definition. They showed a lot of interest in the quality of content and the importance of creating works that reveal artistry, especially in the OTT industry. This finding reflects the characteristics of South Korean culture, where artistry is important in the meaning of content. Although the OTT industry has global characteristics, studies on the identity of the country that supplies the content are necessary, and these results could develop the OTT industry culturally.

Third, we must be able to prepare for changes in the OTT industry. Even students in majors related to OTT cannot obtain the information or capabilities for opportunities to enter the OTT industry unless educational institutions prepare them. Therefore, South Korean educational institutions must cooperate with insightful and capable experts to introduce OTT-related curricula systematically.

### Limitations

This study utilized the Q methodology to classify the subjective perceptions of film and media students regarding the over-the-top (OTT) industry. However, the research process revealed several limitations.

First, since the study only included students from a specific university, the study’s findings are not generalizable to all university students. Future research should expand by converting the derived types into factors for quantitative studies. This approach would allow for research on a larger population, including students from different universities and regions, broadening the scope of the study and enhancing its generalizability. Additionally, through such a process, it may be possible to uncover the causes behind the emergence of each type.

Second, this study only targeted students majoring in areas related to the OTT industry, lacking comparative research with non-majors or general students who consume OTT services. Future studies could address this situation by comparing students not directly involved in the OTT industry to analyze differences in perceptions of OTT content. Moreover, researching industry professionals already active in the OTT field could provide deeper insights by comparing their perceptions with those of students.

Third, the study found that students showed insufficient interest in the OTT industry’s technological aspects, education, and systems. Thus, future research could address this by incorporating expert opinions or conducting more in-depth secondary data analyses. As the technological evolution of the OTT industry and the associated educational changes are crucial, we need additional academic research in this area.

Lastly, this study focused on current perceptions, limiting its ability to analyze changes in perceptions over time. Future research could consider longitudinal studies to track shifts in attitudes and perceptions or cross-sectional studies with different populations to analyze changes across various time points. This approach would deepen the understanding of how this study contributes to developing knowledge in the field.

Based on these limitations and directions for future research, studies on perceptions related to the OTT industry can continue to evolve, providing essential foundational data that could contribute to the positive development of the OTT industry in the future.

## Conclusion

This study sought to reveal the perceptions of university students majoring in film and media production on the over-the-top (OTT) industry, and three types appeared. Type 1 considered the diversity and accessibility of OTT meaningful from the consumer’s perspective, and Type 2 emphasized the need to improve the quality of content from the producer’s perspective. Even though Type 3 thought positively about the OTT industry, they tended to think of movies shown in theaters as their final destination. Based on the study’s results, all types thought that the development of the OTT industry would continue. However, they did not show much interest in areas related to the OTT industry’s technology, education, and systems.

This study presents significant implications regarding film and media students’ perceptions of the OTT industry.

First, it emphasizes the need for an integrated curriculum despite the students’ understanding of OTT platforms’s strengths. OTT platforms produce and distribute content differently from the traditional film industry, and to utilize them effectively, a curriculum that combines technical and business elements is necessary. This study shows the importance of such an integrated educational approach, allowing students to understand the unique characteristics of OTT platforms and contribute to the future development of the film industry through their interaction with these platforms.

Second, the research found that students value the qualitative distinction of OTT content. Since creators and viewers engage with OTT content differently from traditional films or dramas, the criteria for evaluating its quality should also evolve. Therefore, there is a need for clear standards for assessing the quality of OTT content. The study suggests that students believe such standards are necessary to balance content completion, artistry, and commercial success.

Lastly, while students positively assess the rapid development of the OTT industry, they also recognize the need for the parallel growth of the film industry. They believe that OTT platforms and cinemas have a complementary relationship, with the immersive experience offered only by movie theaters remaining essential. This perception reflects the growing understanding that we should not view OTT platforms and cinemas as competitors. Instead, these industries should leverage each other’s strengths to grow together.
